# Plant success: The consequence of the hysteretic responses of leaves to ambient water vapor, carbon dioxide, light intensity, and temperature

**DOI:** 10.1007/s10867-026-09723-y

**Published:** 2026-08-12

**Authors:** David Peak, Michael Frame

**Affiliations:** 1 Physics Department, Utah State University, 4415 Old Main Hill, Logan, UT 84322 USA; 2 Hamden, USA

**Keywords:** Plant success, Hysteresis, Memristor, Crossbar array

## Abstract

Plants account for the vast majority of the biomass on land. They first appear in the fossil record many millions of years ago. Some researchers have proposed that the obvious success of plant-life indicates that plants are “intelligent.” Plant intelligence has been strongly challenged, however, because plants do not possess brain-like tissues similar to electrical neurons, axons, and synapses. The following paper argues that a plant leaf is more properly understood as being a kind of analog computer called a “crossbar array.” The leaf’s computational facility arises from how leaf stomata respond to environmental water vapor, light, carbon dioxide, and heat. Though counterintuitive, a leaf’s success is enhanced by the phenomenon of “stomatal oscillations.”

## Introduction

### Why are plants so extraordinarily able to adapt to environmental variability?

Plants are uniquely successful. They first appear in the fossil record about a half billion years ago [1], and, today, they account for over 80% of all of the biomass on land [2].

Plants display many adaptive behaviors that seem human- or animal-like. This has led some researchers to propose the controversial view that plant success arises because “plants are intelligent” [3].

Arguments for the origins of plant “intelligence” have typically focused on the roles of the electrical or the chemical-reaction properties of plants as potential agents for adaptive behavior. But the conjecture that plant adaptability emerges from electrical or chemical-reaction phenomena has been strongly challenged because there are no obvious plant tissues (i.e., no electrical or chemical neurons, axons, or synapses) that might serve these purposes [4]. It is natural, then, to wonder what could be alternatives to electrical- or chemical-activity that might help explain plant success.

Notably, plant stomata appear in the fossil record roughly contemporaneously with the appearance of plants themselves [5]. Elucidating how the success of plants might be related to their stomata is the aim of this paper.

### Observed hysteresis in plant experiments

The stated “aim” above starts with the phenomenon of leaf water vapor hysteresis.

In a review paper, Zhang et al. identified an unexpected *apparent* ubiquity of observed water vapor hysteresis in plant experiments [6]. In the Zhang et al. paper water vapor hysteresis was reported in ten experiments with different species *done in the field*, performed both by the authors and also by different investigators at different times.

In these experiments, with a leaf in bright light (to open the leaf’s stomata), the water evaporation rate, *E*, was measured as a function of periodically varied *vapor pressure deficit*: i.e., $\Delta w={w}_{L}-{w}_{a},$ where ${w}_{L}$ is the calculated saturated water vapor concentration within the leaf at temperature ${T}_{L}$, and ${w}_{a}$ is the measured ambient water vapor concentration. (Note: many authors, including Zhang et al., refer to plant “evapotranspiration.” To be precise, though, these authors actually only measure the water evaporation from the leaf part; the transpiration part refers to the much slower water transport from the plant roots to the plant leaves.)

In all such experiments leaf temperature was held constant (by adjusting air temperature and light intensity as needed) while ambient humidity was varied. Zhang et al. found that it took 6 h for their stomata to be fully open and 18 h for them to fully closed. Though such a difference between stomatal opening and closing might be evidence of genuine hysteresis, it might also arise from the opening/closing asymmetry associated with the 24-h circadian rhythm of plants in the wild (i.e., where the Zhang et al. observations were made).

To distinguish between genuine hysteresis and the apparent hysteresis reported by Zhang et al. it is useful to aggregate results of experiments in which evaporation rate from leaves is measured as a function of varying vapor pressure deficit.

**Fig. 1:**
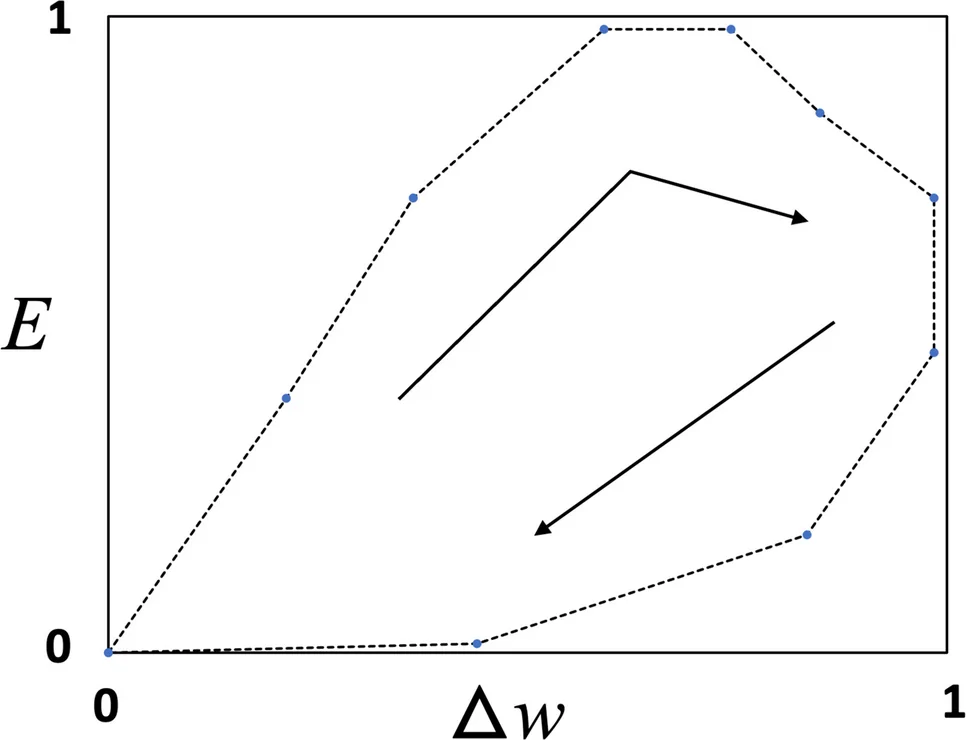
Plot of normalized evaporation rate, E, as a function of normalized vapor pressure deficit, $\Delta \mathrm{w}$

Figure 1 shows an example leaf water vapor hysteresis loop, constructed using data in Zhang et al. [6]. Because the Zhang et al. experiments all have different *E* and Δw ranges it is useful to compare all of the data by setting the minimum and maximum measurements to 0 and 1, respectively, as shown.

The Fig. 1 example has two branches, depicted by the arrows. Along the *E*-increasing branch on the left (where the leaf’s stomata are opening), Δw is increased by decreasing ambient humidity (${w}_{a}$), while along the *E*-decreasing branch (where the leaf’s stomata are closing) on the right, ambient humidity is increased to decrease Δw. The measured time for opening in the Zhang experiment is about 6 h, and for closing is about 18 h. (The lengths of the lines in Fig. 1 do not represent elapsed time.)

Each data point shown in Fig. 1 is acquired at constant conditions of air temperature, carbon dioxide, and ambient light intensity. Note that in the upper right hand corner of Fig. 1, *E* stops increasing and actually decreases even though Δw doesn’t change. That occurs because at very low values of ambient humidity stomatal apertures tend to close even more than just by humidity changes—thus leading to less leaf water loss.

The data points shown in Fig. 1 are average measurements after oscillations have sufficiently subsided. It is argued below that, while they are a challenge to the experimentalist, stomatal oscillations naturally accompany plant success.

Note that hysteresis plots for electric circuits typically have a second loop, corresponding to negative *E* and negative Δw. But negative Δw requires ${w}_{a}$ to be greater than the saturated value of water vapor at the corresponding air temperature, in which case the external vapor would just precipitate out on the leaf surface.

If *E* is thought of as “water current” and Δw is thought of as “applied water voltage,” the ratio Δw/*E* would be equivalent to “water resistance” (i.e., “Ohm’s Law”). The plot in Fig. 1 is nonlinear, so Δw/*E* changes from place-to-place on the diagram. But, because leaf temperature is held constant in the experiments shown, this nonlinear change in water resistance is not associated with the leaf heating or cooling. Rather, it is due to the leaf’s stomatal apertures tending to open more slowly along the *E*-increasing branch and tending to close more rapidly along the *E*-decreasing branch. Though that might seem like a trivial observation, it turns out that such hysteretic opening and closing of leaf stomata is plausibly central to why plants are so successful.

**Fig. 2:**
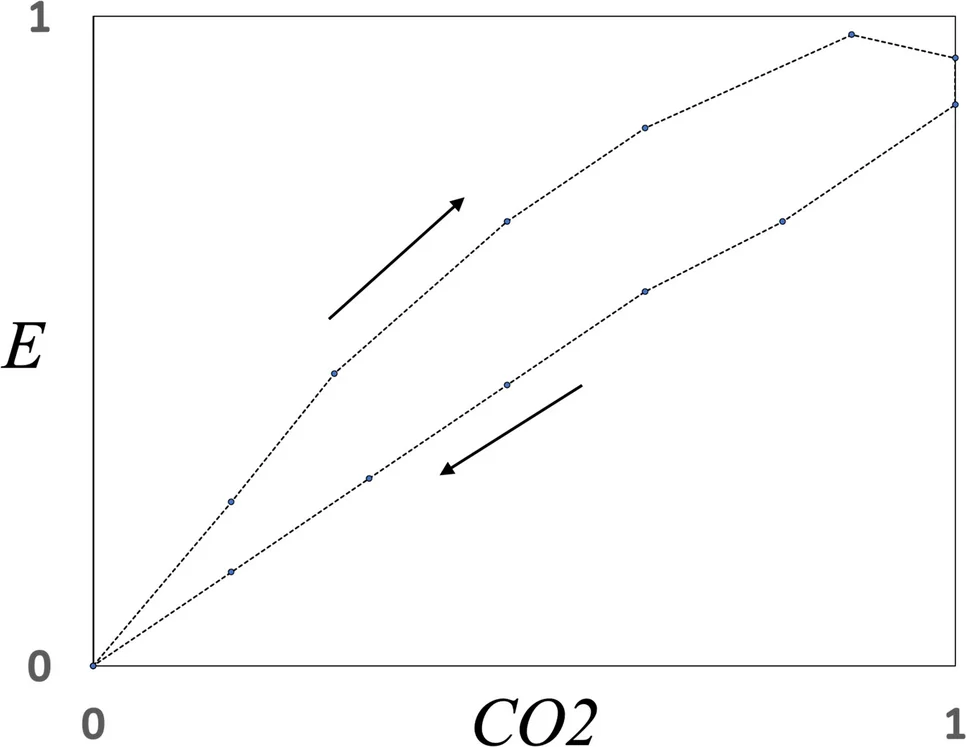
Plot of normalized evaporation rate, E, as a function of normalized ambient carbon dioxide density

Change in ambient humidity is not the only source of leaf water vapor hysteresis. Figure 2*,* for example, shows stomatal response in an experiment where ambient carbon dioxide levels are increased (slow opening along the upper arrow) and decreased (fast closing along the lower arrow) [8]. Here, temperature, ambient water vapor, and light level are held constant.

**Fig. 3:**
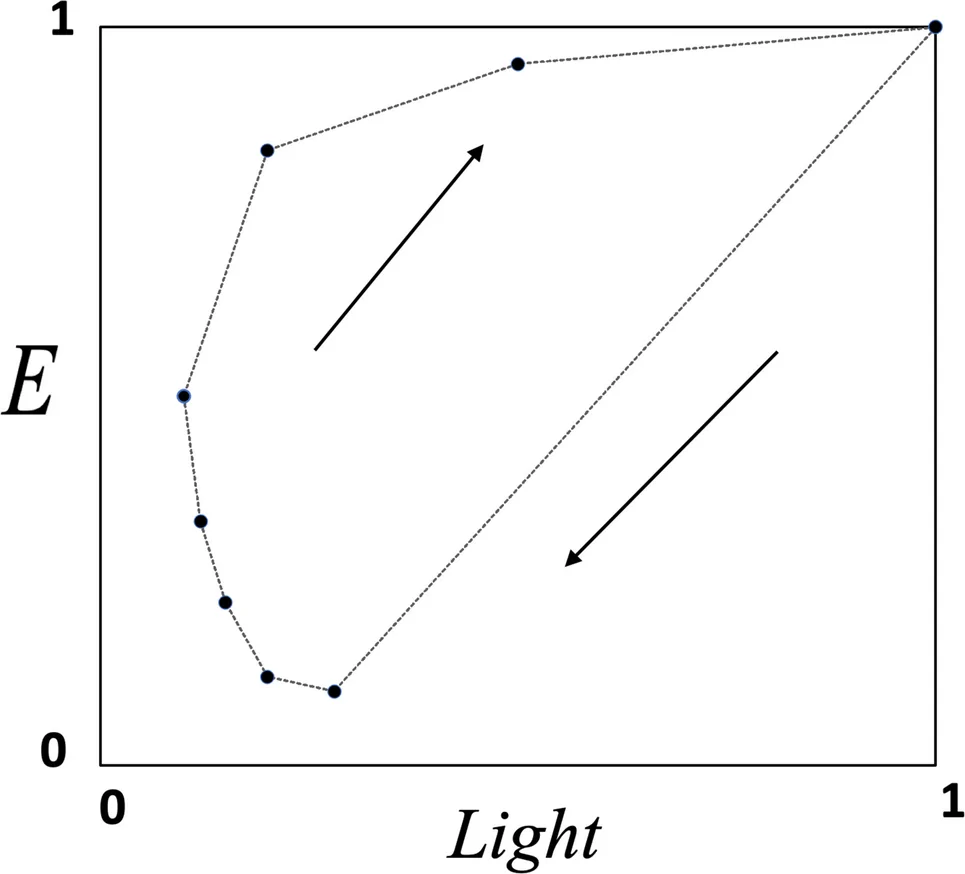
Plot of normalized evaporation rate, E, as a function of ormalized light intensity, light

Figure 3 shows another leaf hysteresis plot in which external carbon dioxide, ambient humidity, and leaf temperature are held constant while light level is changed. In the Figure, the leaf is initially in bright light with open stomata (i.e., in the upper righthand corner). Immediately after reducing the light intensity the stomata rapidly close (though not completely, as in some experiments) shown along the right arrow of the graph. When light is returned to the original level (along the left arrow), the stomata gradually reopen back to the initial condition [9].

When the temperature of the ambient air is rapidly lowered the leaf’s stomata also rapidly close. When the temperature is raised to the starting value, the stomata gradually reopen. The shape of the resulting hysteresis plot is essentially identical to that in Fig. 3 [9, 10].

In summary, a leaf’s stomata periodically open and close whenever water vapor, carbon dioxide, light intensity, or air temperature (and possibly other external inputs) in its environment are individually periodically altered.

These examples are sufficient to make a strong case supporting the proposal that a leaf is like an “analog computer.” To see why that might be so requires a brief foray into contemporary electrical engineering.

## Chua’s Memristor

In 1971, Leon Chua proposed that a fourth fundamental electric circuit element—in addition to the resistor, capacitor, and inductor—should exist [11]. According to Chua, such an electrical element would be a plug of condensed material whose instantaneous electrical resistance would vary depending on how much, and in which direction, an alternating electrical current had recently passed through it. Such a variation would not result from a resistive temperature change, as in an ordinary resistor, but rather to a structural- or phase-change of the material from which the proposed element would be made. Because the resistance of Chua’s fourth element depends on its past conditions, it is therefore a “re*mem*bering *r*e*sistor*.”

An example of Chua’s memristor was finally found experimentally in 2008 [12]. Since then, tens of thousands of papers have appeared in the electrical-science and electrical-engineering literature detailing novel space–time behaviors of circuits that incorporate electrical memristors [13].

Importantly for the proposition that stomata are connected to plant success, Chua has proposed a technically precise definition for an electrical memristor: “a memristor is best defined as any two-terminal device that exhibits a pinched hysteresis loop in the current–voltage plane when driven by any periodic signal that elicits a periodic response of the same frequency” [14]. (A hysteresis diagram is “pinched” at a point where in- and out-lines with similar slopes connect. Examples are the 0,0 points on Figs. 1 and 2 and the 1,1 point on Fig. 3.)

Chua’s definition of an electrical memristor involves: (1) an individual electrical element—a small solid-state plug; (2) an applied alternating electric potential difference; and (3) the resulting alternating electric current. A graph of the current versus applied potential for such a device is “pinched” where the slopes of the applied potential difference and the resulting current change signs.

The water vapor, carbon dioxide, light, and temperature hysteresis examples described above, on the other hand, involve whole leaves (i.e., many stomata), water vapor evaporation, and several different environmental “driving forces.”

In 2023, Peak, Hogan, and Mott published a detailed mathematical model [7] (herein referred to as the *PHM* model), for stomatal dynamical behavior that successfully aggregates a large number of observed plant phenomena into a network of spatially coupled time differential equations. The *PHM* equations involve a leaf’s guard- and epidermal-cells and its mesophyll tissue. When suitably parametrized, the associated mathematical apparatus quantitatively accounts for such ostensibly unrelated observed stomatal phenomena as the: (1) “oscillations in red light are damped in blue light” response; (2) “wrong-way” response to rapidly lowered humidity; (3) “oscillations in darkness” phenomenon; and (4) formation of “stomatal patches” shortly after a leaf in darkness is exposed to bright light. In other words, the *PHM* apparatus reliably describes a variety of observed phenomena shared by many different plant species.

**Fig. 4:**
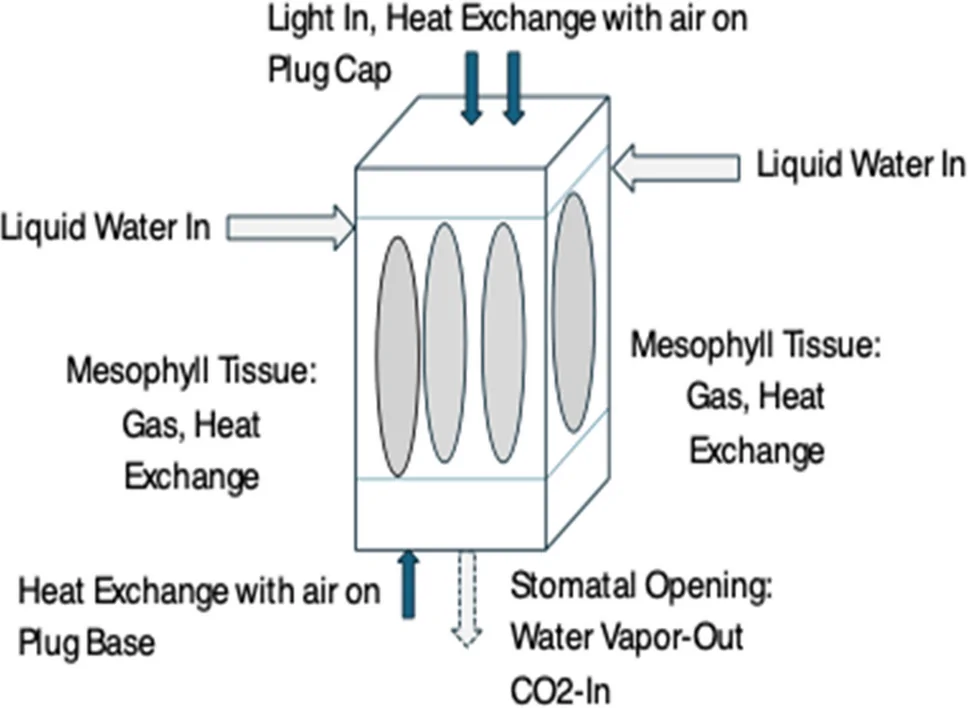
Schematic of a stomatal plug

To see what Chua’s memristors have to do with leaves, it is essential to reprise the concept of a “stomatal plug” introduced in the *PHM* paper. A stomatal plug is defined in *PHM* as a porous columnar volume of mesophyll tissue and intercellular spaces. See Fig. 4.

The top of the column contains liquid water that has entered the leaf from outside, via the leaf’s petiole. The base of the column has guard- and epidermal-cells that together define a variable aperture pore—the plug’s stoma—through which water vapor can escape. Figure 4 indicates various energy and mass exchanges a stomatal plug can engage in with its plug neighbors and with its external environment. Leaves are composed of many such stomatal plugs packed together, one plug for every stomatal opening. Such plugs are connected to one another in species-dependent different ways.

Though stomata in a leaf often have variable spacing and somewhat variable structure, it is reasonable to approximate the stomatal plugs as being identical “on average.” Thus, in Figs. 1, 2, 3 the hysteresis of the whole leaf can be envisioned as arising from the coordinated hysteretic opening and closing of the apertures in the leaf’s “average stomatal plugs.” The plots in Figs. 1, 2, 3 are all “pinched” where the opening and closing segments intersect, and the vapor resistance depends on the branch of the plot. According to Chua’s “best definition” then, (average) stomatal plugs are “water memristors.” While Chua’s electrical memristors are only affected by electric current input, a leaf’s stomatal plug memristors are affected at least by water, carbon dioxide, light, and temperature inputs.

### The electric crossbar

**Fig. 5:**
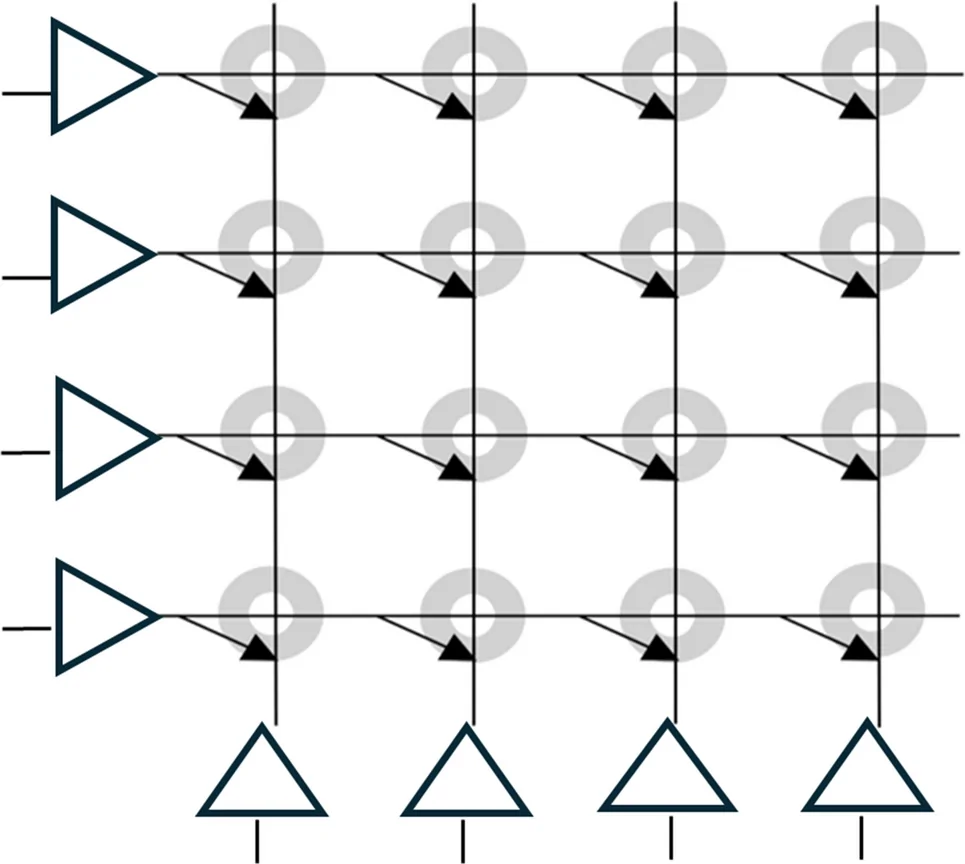
Portion of an electrical crossbar array

Figure 5 is a cartoon representation of an electronic “crossbar array.” In it, the white triangles are transistors and the arrows are electrical memristors. The transistors on the left in the figure transmit electrical information (through the thin wires) from the external world and activate different transistors on the bottom of the array. When done properly, such transistor-to-memristor-to-transistor processes can perform computations. That is, the electrical memristors on the crossbar “compute.” (In 2021, Chua and Volkov [15] published a description of how electric current passing through metal/insulator or plant tissue plugs could behave dynamically as memristors. Though important for describing the time course of the dynamics of a single memristor, [15] says nothing about how computation emerges from an array of interacting memristors—such as a leaf.)

A reason memristor networks have generated so much interest is that the memristors in the network “remember” the current that has recently passed through them. Thus, the result of the computation performed by each memristor is temporarily stored “in place.” This provides a substantial potential advantage over electrical computations involving traditional resistors in which information is stored “in memory,” retrieved and manipulated, and then put back in storage. The latter sequence of events takes time and wastes energy.

Despite their attractiveness, memristor networks have a seemingly deleterious characteristic. Recently, it has been discovered that electrical memristor networks (such as a crossbar array) display aperiodic oscillatory behavior. Such behavior results from small, naturally occurring, effective inductances or capacitances in the memristor circuit [16]. This kind of electrical noise would seem to be detrimental to the intended computation.

But, even more recently it has been discovered that such oscillations in the memristor network have a surprising benefit: they actually reduce the network’s input-to-output electrical noise [17]. In other words, computations on an electric crossbar array are made more reliable because the material from which the array is made has intrinsic oscillations!

### The water crossbar

Figure 5 suggests an approximate structural similarity of a leaf and an electrical crossbar array equipped with electrical memristors. The leaf’s memristors are its stomatal plugs—depicted by the circular rings in Fig. 5. For a leaf, the “in” transistors in the figure represent all of the leaf’s external inputs (light, temperature, carbon dioxide, water vapor, …); the wires represent how adjacent plugs share water and influence each other’s states; the “out” transistors represent all of the responses (e.g., varying its stomatal openings) a leaf can make to its inputs. These “in”/“out” correspondences are akin to the leaf executing a program. This program is obviously important because it determines the leaf’s “success.”

So, is there a source of oscillations in a leaf that might help keep its program reliable? The answer is found in the phenomenon of *stomatal oscillations* [18]. Any credible theory of stomatal behavior must include such oscillations.

The *PHM* paper [7] referred to previously predicts that stomatal oscillations naturally occur in *all* of the various leaf experiments analyzed in that paper. Stomatal oscillations observed in the laboratory are ubiquitous. They are often considered to be the bane of the plant experimentalist. A typical leaf experiment employing a gas-exchange chamber will not yield “useful” (i.e., “all the stomata doing more-or-less the same thing”) data until the amplitudes of these oscillations become small enough to be ignored. Sometimes this might take hours after the start of the experiment, and sometimes it might never occur on a given day.

## Summary conclusion

Common wisdom has been that stomatal oscillations are detrimental to plant success and therefore their existence has been a puzzle. Contrary to this perspective, the argument presented here is that stomatal oscillations can actually help “clean up” the uncertainty in a leaf’s response to its variable environmental input—and are therefore central to why plants are so successful.

## Data Availability

No datasets were generated or analysed during the current study.
